# Is There a Role for Risk-Reducing Bilateral Breast Surgery in *BRCA1/2* Ovarian Cancer Survivors? An Observational Study

**DOI:** 10.3390/curroncol30090567

**Published:** 2023-08-23

**Authors:** Daniela Oliveira, Sofia Fernandes, Isália Miguel, Sofia Fragoso, Fátima Vaz

**Affiliations:** 1 Medical Genetics Unit, Centro Hospitalar e Universitário de Coimbra, 3000-602 Coimbra, Portugal; danielaoliveira@chuc.min-saude.pt; 2 University Clinic of Genetics, Faculdade de Medicina, Universidade de Coimbra, 3000-548 Coimbra, Portugal; 3 Clinical Academic Center of Coimbra, 3004-561 Coimbra, Portugal; 4 Familial Cancer Risk Clinic, Instituto Português de Oncologia de Lisboa Francisco Gentil, 1099-023 Lisboa, Portugal; sffernandes@ipolisboa.min-saude.pt (S.F.); imiguel@ipolisboa.min-saude.pt (I.M.); 5 Medical Oncology Service, Instituto Português de Oncologia de Lisboa Francisco Gentil, 1099-023 Lisboa, Portugal; 6 Molecular Pathobiology Research Unit, Instituto Português de Oncologia de Lisboa Francisco Gentil, 1099-023 Lisboa, Portugal; afragoso@ipolisboa.min-saude.pt

**Keywords:** ovarian cancer, breast cancer, hereditary cancer, *BRCA*, risk-reducing bilateral breast surgery

## Abstract

Background: Risk-reducing surgeries are an option for cancer risk management in *BRCA1/2* individuals. However, while adnexectomy is commonly recommended in breast cancer (BC) survivors, risk-reducing bilateral breast surgery (RRBBS) is controversial in ovarian cancer (OC) survivors due to relapse rates and mortality. Methods: We conducted a retrospective analysis of *BRCA1/2*-OC survivors, with OC as first cancer diagnosis. Results: Median age at OC diagnosis for the 69 *BRCA1/2*-OC survivors was 54 years. Median overall survival was 8 years, being significantly higher for *BRCA2* patients than for *BRCA1* patients (*p* = 0.011). Nine patients (13.2%) developed BC at a median age of 61 years. The mean overall BC-free survival was 15.5 years (median not reached). Eight patients (11.8%) underwent bilateral mastectomy (5 simultaneous with BC treatment; 3 RRBBS) at a median age of 56.5 years. The median time from OC to bilateral mastectomy/RRBBS was 5.5 years. Conclusions: This study adds evidence regarding a lower BC risk after *BRCA1/2*-OC and higher survival for *BRCA2*-OC patients. A comprehensive analysis of the competing risks of OC mortality and recurrence against the risk of BC should be individually addressed. Surgical BC risk management may be considered for longer *BRCA1/2*-OC disease-free survivors. Ultimately, these decisions should always be tailored to patients’ characteristics and preferences.

## 1. Introduction

Women with hereditary breast and ovarian cancer syndrome have an increased risk of developing cancer, mainly breast cancer (BC)—absolute risk > 60% for *BRCA1/2* carriers—and ovarian cancer (OC)—absolute risk of 39–58% for *BRCA1* and 13–29% for *BRCA2* carriers [1]. 

Currently, breast imaging, such as ultrasound, mammography and/or magnetic resonance imaging (MRI), is widely recommended to detect malignant lesions at an early stage in *BRCA1/2* women [1]. However, the most significant approach to reduce BC risk in *BRCA1/2* carriers is risk-reducing bilateral breast surgery (RRBBS) [2]. Some previous reports stated that there was a BC risk reduction of 90 to 95% in *BRCA1/2* women that underwent RRBBS, although no significative reduction in mortality was observed [3]. Likewise, risk-reducing adnexectomy is strongly recommended, typically between 35 and 40 years, to manage OC risk in *BRCA1/2* women [1]. In addition to a profound decrease in OC incidence, risk-reducing adnexectomy also leads to a substantial reduction in all-cause and OC-related mortality [3]. While risk-reducing adnexectomy is still commonly recommended in *BRCA1/2*-BC survivors, RRBBS is controversial in *BRCA1/2*-OC survivors, due to the high relapse rate and mortality associated with OC. In fact, there is a lack of thorough recommendations concerning BC risk and the role of RRBBS in *BRCA1/2*-OC survivors. In this study, we evaluate the incidence of BC after *BRCA1/2*-OC and report our experience with RRBBS in these patients.

## 2. Materials and Methods

All consenting women testing positive for *BRCA1* or *BRCA2* were invited to participate in long-term prospective follow-up in the Familial Risk Clinic of IPO Lisboa. Patients are kept under surveillance until death, loss to follow-up or consent withdrawal. For this study, patients with OC as first cancer diagnosis and a *BRCA1/2*-positive test between January 2000 and August 2022 were selected. Women who had had another cancer before OC were excluded. Data before testing were retrospectively collected from available clinical reports. The start of the follow-up period was defined as the date of OC diagnosis. The overall and BC-free survival were calculated using the Kaplan–Meier method. The log-rank test was used to compare survival and BC incidence between different groups. Overall survival was considered as the time from OC diagnosis to the time of death, whereas BC-free survival was defined as the time from OC to BC diagnoses. Statistical analysis was conducted using SigmaPlot software, version 15.0.

## 3. Results

Over a period of 22 years, a total of 69 women, from 63 different families, were diagnosed with *BRCA1/2*-OC. The median age at OC diagnosis was 54 years (range: 18–85 years). Most patients for whom data were available were diagnosed with epithelial serous OC (75.9%) and at a III or IV FIGO stage (73.7%). Regarding molecular testing, 33 (47.8%) individuals were identified with a germline *BRCA1* variant and 36 (52.2%) with a germline *BRCA2* variant. Of the 36 *BRCA2* patients, 9 (25%) had the founder variant of Portuguese origin *BRCA2*:c.156_157insAlu. Among the 69 patients, 55 (79.7%) had at least one relative with BC, while 14 (20.3%) had no known relatives diagnosed with BC. Among those with positive family history of BC, 38 (69.1%) had an affected first-degree relative (Table 1). In the subgroup of nine patients who developed BC, eight (88.9%) reported positive family history, and only one (11.1%) patient had no family history of BC. Among those eight with a positive family history of BC, half had an affected first-degree relative (Table 2).

The median duration of follow-up for all patients since OC diagnosis was 6 years (range: 1–22 years). In this group, there were a total of 35 deaths from all causes (all-cause mortality rate: 50.7%) throughout the follow-up period. Death occurred at a median age of 59 years (range: 40–89 years), at a median of 4 years (range: 1–16 years) after OC diagnosis, and, in 94.3% of the cases (33 patients), within the first 10 years of follow-up. For the entire cohort, the median overall survival was 8.0 years (mean: 11.6 years), being significantly higher for *BRCA2*-OC patients (median: not reached; mean: 14.3 years) than for *BRCA1*-OC patients (median: 5 years; mean: 8 years) (*p* = 0.011). 

**Figure 1:**
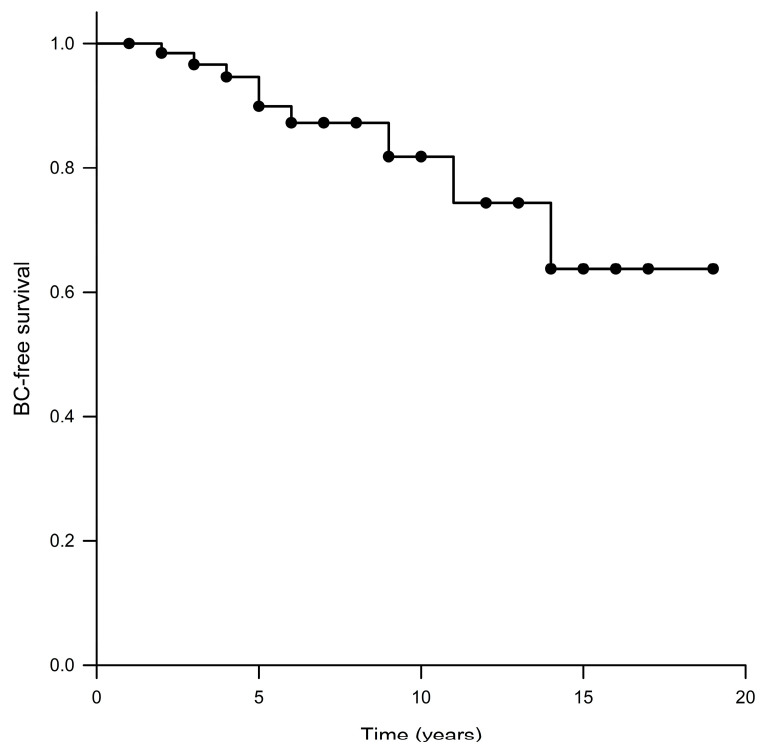
Kaplan–Meier analysis of BC-free survival.

Further, one of the 69 patients was lost to follow-up more than two years before death, so she is not considered when assessing BC (or other cancer types) risk in this cohort. A total of nine (13.2%) patients developed BC after the OC at a median age of 61 years (range: 44–68 years). The median BC-free survival could not be calculated via Kaplan–Meier survival analysis because of the small number of patients who were diagnosed with BC after OC, with the mean BC-free survival in the total population being 15.5 years (Figure 1). The difference in BC-free survival between *BRCA1*-OC women (median: not reached; mean: 12.9 years) and *BRCA2*-OC women (median: not reached; mean: 15.9 years) did not reach statistical significance (*p* = 0.440). The difference in the overall survival between *BRCA1/2*-OC women with BC (median: not reached; mean: 16.4 years) and *BRCA1/2*-OC women without BC (median: 8.0 years; mean: 9.9 years) was also not statistically significant (*p* = 0.107).

All diagnosed BCs were unilateral, and two of them were triple-negative (both *BRCA1* patients). Of the nine patients with BC, five (55.6%) had a *BRCA1* variant, whereas four (44.4%) had a *BRCA2* variant (two with the Portuguese founder variant). Three out of the nine *BRCA1/2*-OC women with BC died at a median age of 51 years (range: 50–61 years) and at a median time of 7 years after the OC diagnosis (range: 5–9 years). The cause of death of these three patients was unrelated to BC: 1—ovarian cancer progression; 2—refractory leukemia; 3—overdose in a patient in remission of both cancers. The characterization of BC diagnosed in our cohort is detailed in Table 2. 

Five (7.4%) patients underwent bilateral mastectomy in a unilateral BC context (Table 3). Bilateral mastectomy was performed at a median age of 54 years (range: 44–69 years) and at a median of 6 years (range: 2–15 years) after the OC diagnosis. Three (4.4%) patients, without personal history of BC, were submitted to RRBBS at a median age of 58 years (range: 55–61 years) (Table 3). The median time from OC diagnosis to RRBBS was 5 years (range: 3–15 years).

In this cohort, five other cancers were diagnosed during the follow-up period—a squamous cell carcinoma of the tongue at the age of 51 (one patient), a synchronous high-stage serous carcinoma of the endometrium at the age of 75 (one patient) and basal cell carcinomas of the skin at ages of 59 and 72 (two patients). One patient was diagnosed with acute myeloid leukemia, secondary to chemotherapy for ovarian cancer, at the age of 60 and BC at the age of 61.

## 4. Discussion

In this study, we observed an incidence of 13.2% of BC in a cohort of 68 *BRCA1/2*-OC women during a median follow-up period of 6 years. The mean BC-free survival in the total population was 15.5 years (median: not reached), with no significant difference for *BRCA1*-OC, as compared to *BRCA2*-OC patients. While we observed a significantly higher overall survival for *BRCA2*-OC patients as compared with *BRCA1*-OC patients, the difference in overall survival between *BRCA1/2*-OC women with BC and *BRCA1/2*-OC women without BC was not found to be statistically significant.

The incidence of BC in our cohort was slightly higher than that reported in previous studies, such as that described by Vencken et al. [4], who identified 8 primary BCs in 79 *BRCA1/2*-OC women (10.1%) during a mean period of 6.7 years, and by Domchek et al. [5], who reported 11% of BCs (18 patients) in a group of 164 during a mean follow-up of 5.8 years. Similarly, Gangi et al. [6] described an incidence of 8.9% of BC (12 women) in 135 patients with a mean follow-up period of 6.6 years, and Fong et al. [7] identified 8.3% (16 patients) in 192 *BRCA1/2*-OC women. More recently, a larger cohort of 502 patients was characterized by Safra et al. [8], who reported a lower incidence (6.2%) of BC in 502 *BRCA1/2*-OC women, with a median follow-up of 5.0 years. The small number of *BRCA1/2*-OC women included in most of the studies, including our own, is a limitation regarding conclusions about the incidence of BC in this specific population. However, caution should also be taken when drawing conclusions, as inclusion criteria and mutational *BRCA1/2* patterns differ among these studies. For example, Safra et al. [8] included women with BC and other cancers diagnosed prior to OC (representing 17.5% and 1.6% of the cohort, respectively) in their cohort. In our study, any type of cancer diagnosed before OC was an exclusion criterion. Another note that must be emphasized is that women who underwent RRBBS were maintained in our cohort, even after prophylactic surgery. Even after RRBBS, there is a remaining BC risk, and, in our registry, patients are kept in surveillance until death, are lost to follow-up or withdraw their consent.

Regarding mutational *BRCA1/2* patterns, our study is the first where the numbers of *BRCA2*-OC women are higher than *BRCA1*-OC patients (*BRCA2*: 52.2% vs *BRCA1*: 47.8%). In all previous studies, *BRCA1*-OC patients represented more than 70% of the cohort [4,5,6,7,8]. As we discuss below, data are conflicting regarding *BRCA1* and *BRCA2* as biomarkers for better survival when compared with sporadic OC. However, a relevant finding in our study is the increased overall survival observed in the *BRCA2*-OC subgroup when compared to *BRCA1*-OC patients (14.3 years vs. 8 years, *p* = 0.011). This observation was previously described in a pooled analysis of 26 studies [9].

Vencken et al. [4] reported a BC risk of 3%, 6% and 11% in *BRCA1/2*-OC survivors in the following 2, 5 and 10 years after OC diagnosis. The same study reported a significantly higher BC risk in unaffected variant carriers during the same follow-up period (6%, 16% and 28%, respectively) [4]. Domchek et al. [5] also reported a less-than-10% risk of developing BC in the 10-year follow-up period (12% for *BRCA1* carriers and 2% for *BRCA2* carriers). In a more recent study, McGee et al. [10] reported a risk of 7.8% of developing BC in a 10-year interval, conditional on OC survival and other causes of death. Despite the fact that data are still limited and larger studies are needed, *BRCA1/2*-OC survivors appear to have a significantly lower risk of developing BC after OC than unaffected individuals. Previous studies suggested several reasons for the apparent lower BC risk in *BRCA1/2*-OC survivors compared to unaffected women. One of the reasons is the premature termination of ovarian function due to salpingo-oophorectomy, usually performed in an OC treatment context [4,5,6]. In line with that, previous studies reported that risk-reducing adnexectomy reduces the risk of BC in *BRCA1/2* patients, mainly if performed at a premenopausal age [4,5,11,12]. Another aspect that is likely to contribute to a lower rate of BC in this subgroup is the effect of the therapy for OC. Some authors argue that platinum-based chemotherapy usually used for OC treatment could contribute to eradicating submicroscopic breast disease, leading to a lower number of BCs in these patients [4,5,6]. 

Although there are conflicting data in the literature, some studies reported that carrying a *BRCA1* or *BRCA2* variant leads to better responses to both platinum- and non-platinum-based chemotherapies, as well as better progression-free and overall survival in OC patients [13]. Nonetheless, McLaughlin et al. [14] demonstrated that *BRCA1* or *BRCA2* variants could be an advantage in OC patients to short-term survival but not to long-term survival. Although there is a trend for increasing OC survival due to recent advances in therapeutic approaches, OC 5-year and 10-year survival rates remain poor (63% and 35%, respectively) [4]. In our study, we report a mortality rate of 50.7% at a median age of 59 years, which is similar to those previously reported (53.2%, 51.1%, 40.7% and 36.3%) [4,6,8,10]. Moreover, most patients in our cohort (74.6%) were diagnosed with a high-stage OC, which is comparable to the 76% reported in Vencken et al. [4] and the most prevalent stage IIIC in Gangi et al. [6]. Currently, patients’ outcome is essentially determined by OC mortality rate and the risk of relapse, mainly during the first years after diagnosis. Noteworthily, 94.3% of deaths in our cohort occurred within the first 10 years of follow-up after OC diagnosis. We registered three deaths among *BRCA1/2*-OC women with BC but none were linked to the diagnosis of BC. Similarly, Domchek et al. [5] and Safra et al. [8] also stated that none of the deaths in their cohorts of women with OC and BC were related to BC. In the Fong et al. [7] study, only one patient died of BC.

Taking all data into consideration, we propose that BC after *BRCA1/2*-OC would still require specific surveillance, especially in those women who have better prognosis. Based on simulation studies, McGee et al. [10] concluded that the risk of death from BC after OC is about 1%, and breast MRI screening and RRBBS will have a very small impact on survival. For example, among all *BRCA1/2* women diagnosed with stage III/IV OC at the age of 50, breast MRI screening and RRBBS will reduce, by 1% and 2%, respectively, the chance of dying by the age of 80. However, these effects could be greater if OC was diagnosed at an early age or at lower stages, leading several authors to propose that RRBBS or breast MRI screening should be recommended to all patients diagnosed with stage I or II OC and those patients with stage III or IV OC diagnosed at or before the age of 50 and surviving at least 10 years without relapse [10]. It is of note that in our study, eight patients underwent BC risk-reducing surgery, but for five of these patients, surgery was decided in the context of a BC diagnosis and regarding contralateral BC risk reduction. 

We are aware that several limitations of this study, particularly the small cohort and the lack of a control group, should be considered when conclusions are discussed. Data regarding individual treatment and OC relapse were also not included in the current discussion. However, this study adds data to the discussion regarding the risk of developing BC in a population of *BRCA2*-enriched OC survivors. With recent advances in treatment, the number of *BRCA1/2*-OC survivors may increase in the near future, and this information may help clinicians to provide more accurate counseling regarding BC risk and risk management options to these patients. We are looking forward to in-depth future collaborative studies, including larger cohorts, so as to obtain more robust recommendations for this subgroup.

## 5. Conclusions

During the early period after OC diagnosis, OC mortality and recurrence rates are significantly high, and BC risk appears to be lower than in unaffected *BRCA1/2* individuals. With that in mind, invasive BC risk management could bring an inappropriate burden without significant benefits to these patients. However, with the positive survival impact of new therapeutic advances, we expect a rising number of *BRCA1/2*-OC survivors with health professionals having to face the dilemma of RRBBS in the context of a potentially life-limiting OC diagnosis. We propose a comprehensive analysis of the competing risks of OC mortality and OC recurrence against the risk of BC that should be individually addressed, particularly in those patients with longer disease-free survival. Ultimately, decisions regarding preventive measures should always be tailored to patients’ characteristics and preferences.

## Figures and Tables

**Table 1:** Characterization of the cohort.

**CHARACTERISTIC**	
Number of patients	69
Number of families	63
**MOLECULAR RESULT**	
*BRCA1*	33 (47.8%)
*BRCA2*	36 (52.2%)
*BRCA2*:c. c.156_157insAlu	9 (25%)
**OVARIAN CANCER**	
Median age [range]	54 y [18–85 y]
FIGO stage	
I	11 (18.0%)
II	5 (8.2%)
III	34 (55.7%)
IV	11 (18.0%)
Unknown	8
Histology	
Epithelial serous	44 (75.9%)
Epithelial endometrioid	3 (5.2%)
Epithelial mucinous	1 (1.7%)
Epithelial transitional cell	1 (1.7%)
Mixed epithelial serous and endometrioid	2 (3.4%)
Mixed epithelial serous and transitional cell	1 (1.7%)
Poorly differentiated	6 (10.3%)
Unknown	11
**NUMBER OF DEATHS (all causes)**	35 (50.7%)
Median age [range]	59 y [40–89 y]
Median time after OC [range]	4 y [1–16 y]
Death within the first 10 years of follow-up	33 (94.3%)
**FAMILY HISTORY OF BC**	
Positive—all known relatives	55 (79.7%)
First-degree relatives	38 (69.1%)
Negative	14 (20.3%)

**Table 2:** Characterization of BC diagnosed in the cohort.

**BREAST CANCER**	9 (13.2%)
Median age [range]	61 y [44–68 y]
Median time after OC [range]	5 y [2–14 y]
**RECEPTORS**	
ER and PR negative	2 (22.2%)
ER and/or PR positive	7 (77.8%)
Her2 negative	7 (77.8%)
Triple negative	2 (22.2%)
**FAMILY HISTORY OF BC**	
Positive-all known relatives	8 (88.9%)
First-degree relatives	4 (50.0%)
Negative	1 (11.1%)

**Table 3:** Characterization of bilateral mastectomy and RRBBS in the cohort.

**BILATERAL MASTECTOMY**	5 (7.4%)
Median age [range]	54 y [44–69 y]
Median time after OC [range]	6 y [2–15 y]
**RRBBS**	3 (4.4%)
Median age [range]	58 y [55–61 y]
Median time after OC [range]	5 y [3–15 y]

## Data Availability

Data are contained within the article.
